# Structural and Biochemical Bases for the Inhibition of Autophagy and Apoptosis by Viral BCL-2 of Murine γ-Herpesvirus 68

**DOI:** 10.1371/journal.ppat.0040025

**Published:** 2008-02-01

**Authors:** Bonsu Ku, Jae-Sung Woo, Chengyu Liang, Kwang-Hoon Lee, Hyang-Suk Hong, Xiaofei E, Key-Sun Kim, Jae U Jung, Byung-Ha Oh

**Affiliations:** 1 Division of Molecular and Life Sciences, Center for Biomolecular Recognition, Pohang University of Science and Technology, Pohang, Kyungbuk, Korea; 2 Department of Microbiology and Molecular Genetics and Tumor Virology Division, New England Primate Research Center, Harvard Medical School, Southborough, Massachusetts, United States of America; 3 Biomedical Research Center, Korea Institute of Science and Technology, Seoul, Korea; Oregon Health and Science University, United States of America

## Abstract

All gammaherpesviruses express homologues of antiapoptotic B-cell lymphoma-2 (BCL-2) to counter the clearance of infected cells by host antiviral defense machineries. To gain insights into the action mechanisms of these viral BCL-2 proteins, we carried out structural and biochemical analyses on the interactions of M11, a viral BCL-2 of murine γ-herpesvirus 68, with a fragment of proautophagic Beclin1 and BCL-2 homology 3 (BH3) domain-containing peptides derived from an array of proapoptotic BCL-2 family proteins. Mainly through hydrophobic interactions, M11 bound the BH3-like domain of Beclin1 with a dissociation constant of 40 nanomole, a markedly tighter affinity compared to the 1.7 micromolar binding affinity between cellular BCL-2 and Beclin1. Consistently, M11 inhibited autophagy more efficiently than BCL-2 in NIH3T3 cells. M11 also interacted tightly with a BH3 domain peptide of BAK and those of the upstream BH3-only proteins BIM, BID, BMF, PUMA, and Noxa, but weakly with that of BAX. These results collectively suggest that M11 potently inhibits Beclin1 in addition to broadly neutralizing the proapoptotic BCL-2 family in a similar but distinctive way from cellular BCL-2, and that the Beclin1-mediated autophagy may be a main target of the virus.

## Author Summary

In higher animals, defective or surplus cells are removed by a process known as apoptosis. On the other hand, defective or damaged cellular components are removed by a process known as autophagy. These two destructive processes are indispensable for the survival and development of an organism. While apoptosis is known as a central host defense mechanism that removes virus-infected cells, the role of autophagy against viral infection has recently emerged. Many viruses express an armory of viral proteins that counteract cell death–mediated innate immune control. One such protein is a homologue of the cellular BCL-2 protein that suppresses apoptosis through inhibitory binding to apoptosis-promoting proteins. Murine γ-herpesvirus 68 also encodes a viral BCL-2, known as M11. In this study, we quantitatively measured the binding affinity of M11 for its potential cellular targets, including ten different proapoptotic proteins and the proautophagic protein Beclin1. We found that M11 neutralizes the proapoptotic proteins broadly rather than selectively to suppress apoptosis. Surprisingly, M11 bound to Beclin1 with the highest affinity, which correlated with its strong antiautophagic activity in cells. These data suggest that M11 suppresses not only apoptosis but also autophagy potently, which ultimately contributes to the viral chronic infection.

## Introduction

Gammaherpesviruses are DNA viruses comprising a subfamily of the *Herpesviridae*. These viruses, including Epstein-Barr virus, Kaposi's sarcoma-associated herpesvirus (KSHV) and murine γ-herpesvirus 68 (γHV68), are etiological agents of lymphoid and epithelial tumors in human or animals [1,2]. All γ-herpesviruses encode at least one homologue of the cellular apoptosis inhibitor BCL-2, and expression of these viral BCL-2 genes prevents cell death under various apoptosis-inducing conditions [3–6]. In particular, critical roles of the BCL-2 homologue of γHV68 have been determined by *in vitro* and *in vivo* studies in the pathogenesis of the γHV68 virus. The protein, known as and referred to as M11 here, protected cells from undergoing apoptosis induced by a variety of factors, such as dexamethasone treatment, γ-ray irradiation, CD3ɛ ligation [7], tumor necrosis factor treatment [8,9], Fas ligation [9], and Sindbis virus infection [10]. Furthermore, the protein contributed to latency establishment [11] and was required for efficient reemergence from latency as well as persistent replication during chronic infection of the virus in immunocompromised mice lacking interferon-γ [12]. These data indicate that removal of virus-infected cells by cell death is a central host defense mechanism against viral infection, and viral BCL-2 proteins play a crucial role in the course of viral replication by inhibiting the death of host cells [1,13,14].

The BCL-2 family proteins, which are commonly known as positive or negative regulators of apoptosis, are characterized as containing up to four conserved stretches of amino acids, known as BCL-2 homology (BH) domains [15,16]. BH3-only proteins, a group of proapoptotic BCL-2 family including BIM, BAD, PUMA and Noxa, sense prodeath signals and ultimately activate the downstream proapoptotic members BAX and BAK [17,18]. Activated BAX and BAK cause mitochondrial dysfunction and lead to the release of proapoptogenic molecules, such as cytochrome *c* [19,20]. The interactions between such proapoptotic BCL-2 family members and the antiapoptotic members, such as BCL-2 and BCL-X_L_, are the crucial events in controlling or promoting apoptosis [15,16]. These interactions are mediated by the BH3 domain of the proapoptotic members that binds to a site known as the BH3-binding groove in the antiapoptotic members [21,22].

In addition to their critical roles in the regulation of apoptosis, the BCL-2 family proteins have emerged as regulators of autophagy, a catabolic process that plays crucial roles in cell survival, tumor suppression, and innate immune defense against intracellular pathogens by degrading cytoplasmic components through lysosomal pathway [23–25]. The leading work was the identification of Beclin1 as a BCL-2-interacting protein [26]. A series of subsequent studies showed that Beclin1 promotes autophagy as a component of a multiprotein complex containing class III phosphatidylinositol 3-kinase (PI(3)KCIII) and UV irradiation resistance-associated gene (UVRAG) [27–29], and that BCL-2 negatively regulates the autophagy-promoting activity of Beclin1 [30], while the BH3-only protein BAD plays an autophagy-stimulatory function by disrupting the interaction of BCL-2 or BCL-X_L_ with Beclin1 [31]. While Beclin1 exhibits no overall sequence homology with the BCL-2 family proteins, the recently reported structure of BCL-X_L_ in complex with a Beclin1 peptide revealed the presence of a novel BH3 domain in Beclin1 that binds to the BH3-binding groove of BCL-X_L_ [32]. As observed with the cellular kin, expression of the viral BCL-2 protein of KSHV or γHV68 significantly inhibits autophagy in a Beclin1 binding-dependent manner [28,30], suggesting that these two viral BCL-2 proteins may function as autophagy inhibitors as well as apoptosis inhibitors.

In this study, we determined the structure of M11 in complex with a 50-residue Beclin1 fragment containing its BH3-like domain. Ensuing analyses revealed that M11 binds Beclin1 significantly more tightly than cellular BCL-2 through tighter hydrophobic interactions. Consistently, transiently expressed M11 inhibited autophagosome formation more efficiently than cellular BCL-2. We also quantified the interactions of M11 with the BH3 peptides derived from the apoptosis mediators BAX and BAK and the eight upstream BH3-only proapoptotic molecules BAD, BIK, BIM, BID, BMF, PUMA, Noxa and Hrk. The binding affinity of M11 was highest for Beclin1 and fairly high for BAK, BIM, Noxa, BID, BMF and PUMA, but comparatively low for BAX and Hrk. In the observed affinity profile, M11 is distinctively different from cellular BCL-2 and also from M11L, a virulence factor of Myxoma virus and a structural mimic of BCL-2 that acts primarily by sequestering BAX and BAK [33]. These data suggest that M11 robustly inhibits the Beclin1-dependent autophagy and broadly neutralizes the proapoptotic BCL-2 family to subvert the host antiviral responses.

## Results

### Interaction of Beclin1 with M11

**Figure 1:**
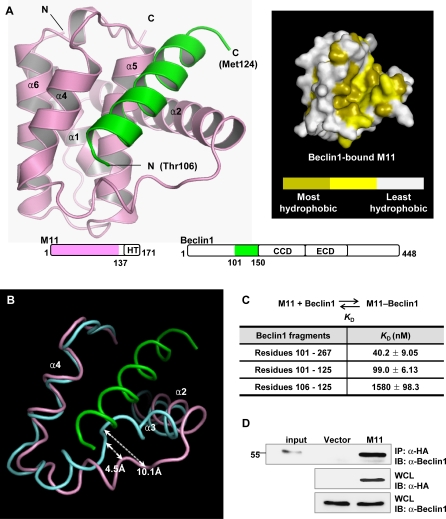
Structural and Binding Analyses of the M11–Beclin1(101–150) Complex (A) Ribbon drawing (left) and surface presentation (right). M11 is in pink, while the Beclin1 helix is in green. The pink and green regions on the primary sequence diagrams indicate the fragments of the M11 and Beclin1 used for the structure determination. “HT”, “CCD” and “ECD” denote hydrophobic tail, coiled-coil domain and evolutionarily conserved domain, respectively. Only 19 amino acids of Beclin1 exhibited well-defined electron density. A surface presentation of M11 with the omission of the Beclin1 helix shows that the BH3-binding groove is predominantly hydrophobic. The surface coloring scheme is as follows: olive for Val, Leu, Ile, Phe, Trp, Met, and Ala; yellow for Cys, Gly, Tyr, and Pro; gray for other amino acids. (B) Large conformational change of M11 induced by the Beclin1 binding. Only the BH3-binding groove region of M11 is shown for clarity. Beclin1(101–150)-bound M11 (pink) and free M11 (cyan) are superposed. The bound Beclin1 peptide is in green. Helix α3 of M11 undergoes a pronounced conformational change. The arrows indicate the movements of the Cα atoms of Asp59 and Tyr60 in M11. (C) ITC analysis. The measurements were carried out by titrating 0.1 mM of M11 into 5 μM of the indicated Beclin1 fragments. The *K*_D_ values were deduced from curve fittings of the integrated heat per mol of added ligand and summarized in the table. (D) M11 interacts with endogenous Beclin1. NIH3T3 cells were transfected with HA-tagged M11 and whole cell lysates were used for immunoprecipitation with anti-HA followed by immunoblotting with anti-Beclin1

Mouse Beclin1 is composed of 448 amino acids. By coexpression test, we found that mouse Beclin1 fragment consisting of residues 101–150, which spans the structurally defined BCL-2-binding region consisting of residues 105–125 (corresponding to residues 107–127 of human Beclin1 [32]), formed a tight complex with M11 lacking the C-terminal hydrophobic tail. The protein in complex with Beclin1(101–150) was crystallized and its structure was determined to 2.3 Å resolution (Table 1). Residues 106–124 of Beclin1 form an α-helix and bind M11 at an extended hydrophobic surface cleft corresponding to the BH3-binding groove of BCL-X_L_ [7] (Figure 1A). In the crystal, the N-terminal five and the C-terminal 26 residues of the Beclin1(101–150) peptide were disordered. The binding of Beclin1(101–150) induces a conformational change of M11 to reshape the BH3-binding groove (Figure 1B). Residues 53–55, a loop segment tailing from α2 in free M11, form an additional helical turn of α2 in Beclin1(101–150)-bound M11 (Figure 1B). In addition, α3 and the following segment undergo a significant conformational transition that involves the translocation of several residues by a distance of 4–10 Å (Figure 1B).

**Table 1:**
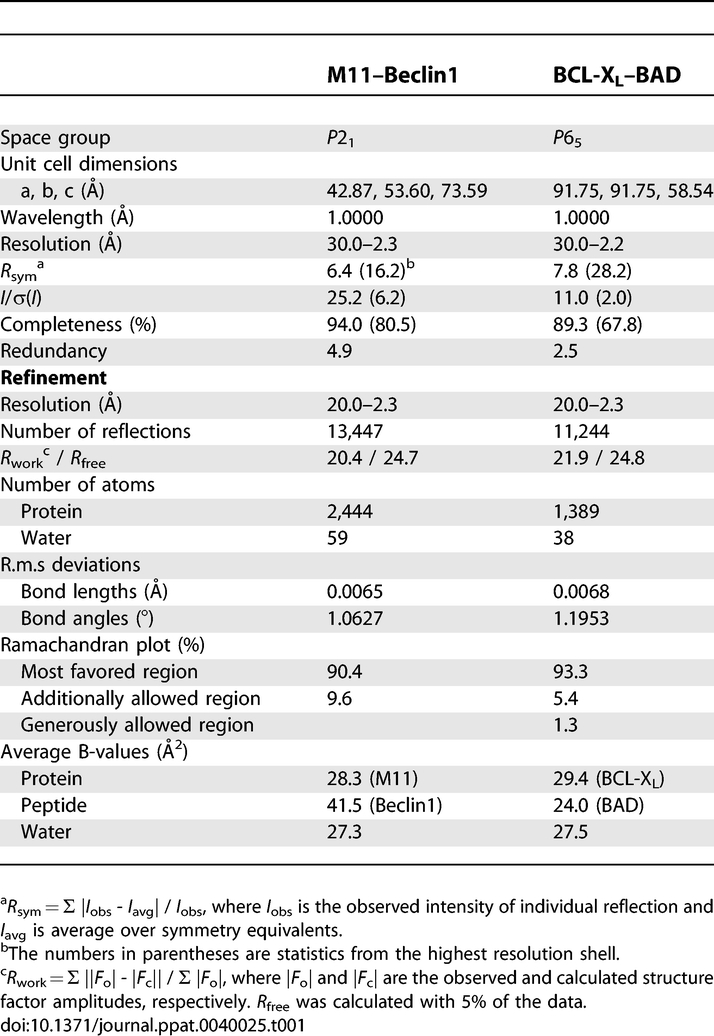
Data Collection and Structure Refinement Statistics

In order to test whether the crystal structure reflects the interaction of Beclin1 with M11 in solution and to determine the strength of their interaction, we performed a quantitative binding analysis using isothermal titration calorimetry (ITC) (Figure 1C and Table S2). We employed a Beclin1 fragment containing residues 101–267 (referred to as Beclin1(101–267)), since this fragment was expressed as a soluble form in E. coli while Beclin1(101–150) was not. This large Beclin1 fragment bound to M11 very tightly with an apparent dissociation constant (*K*_D_) of 40 nM (Figure 1C). Similar binding affinity (*K*_D_ of 99 nM) was observed with a synthetic Beclin1(101–125) peptide (Figure 1C). In contrast, a shorter Beclin1 fragment composed of residues 101–116 exhibited no sign of interaction with the protein (not shown). Unexpectedly, a synthetic Beclin1(106–125) peptide showed quite low binding affinity (*K*_D_ of 1.6 μM) for M11 (Figure 1C), suggesting that residues 101–105 of Beclin1 constitute an important piece in the interaction of Beclin1 peptide with M11, although these five residues were disordered in the crystal and thus are not likely to interact with M11. It was previously shown that residues 140–144 and 161–164 of a BAD peptide contribute to the binding affinity by increasing the helical propensity of the peptide rather than by interacting with BCL-X_L_ [34]. Similarly, a circular dichroism (CD) spectroscopic analysis showed that the Beclin1(101–125) peptide has considerably higher helical contents (29.6%) compared with the Beclin1(106–125) peptide (17.0%) in 30% trifluoroethanol (TFE) solution (Figure S1). The data supports the idea that residues 101–105 of Beclin1 promote the binding of the Beclin1(101–125) peptide to M11 by increasing the helical propensity of the following segment. Conclusively, M11 binds Beclin1 with potently high affinity, and residues 101–125 of Beclin1 compose the minimal region sufficient for binding to M11. In a reflection of the observed potent interaction, we could easily detect the interaction between transiently expressed full-length M11 and endogenous Beclin1 in NIH3T3 cells (Figure 1D).

### Comparison with BCL-X_L_–BAD Complex

**Figure 2:**
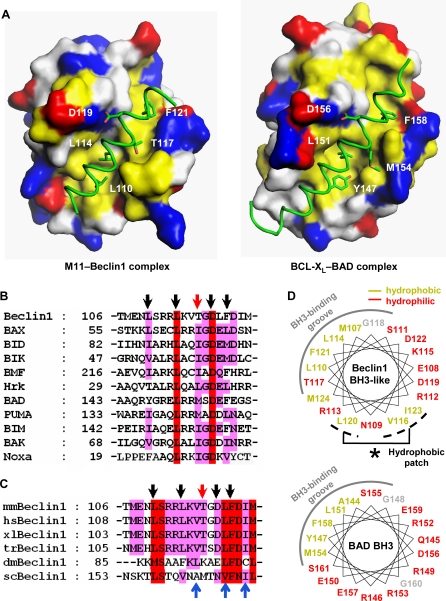
Beclin1 Has a BH3-Like Domain Containing an Atypical Threonine and an Exposed Hydrophobic Patch (A) A structural comparison of the M11–Beclin1(101–150) (left) and the BCL-X_L_–BAD complexes (right). M11 and BCL-X_L_ are shown as surface models. The Beclin1 and BAD residues shown in sticks correspond to the five BH3 residues that are critical for the interactions with antiapoptotic BCL-2 family members [21]. They occupy equivalent positions at the BH3-binding groove in the two structures. The surface coloring scheme is as follows: yellow for Val, Leu, Ile, Tyr, Phe, Trp, Met, and Ala; blue for Lys, Arg, and His; red for Glu and Asp; gray for other amino acids. (B) Sequence comparison of the BH3-like domain of mouse Beclin1 with various BH3 domains. Conserved residues are highlighted by red or pink columns. The arrows indicate the five BH3 residues shown in (A). Of these, Thr117 of Beclin1 (red arrow) is not conserved. (C) Sequence alignment. The BH3-like domains of Beclin1 orthologues are aligned (mm, mouse; hs, human; xl, Xenopus laevis; tr, Takifugu rubripes; dm, Drosophila melanogaster; sc, Saccharomyces cerevisiae). The arrows at the top indicate the BH3 residues shown in (A). These residues are highly conserved throughout species, except for Thr117 of mouse Beclin1, which is conserved only in the vertebrates. The conserved hydrophobic residues of Beclin1 exposed in the structure are indicated by the blue arrows at the bottom. (D) α-helical wheel representation. The Beclin1 α-helix bound to M11 is compared with the BAD α-helix bound to BCL-X_L_. The Beclin1 helix has a hydrophobic patch (indicated by an asterisk) on the opposite side of the BH3-binding groove, unlike the BAD helix.

Cellular antiapoptotic BCL-2 family members share high sequence homology in the BH1, BH2 and BH3 domains, which compose the common and characteristic BH3-binding groove [35]. At a glance, the intermolecular interaction between M11 and Beclin1(101–150) resembled the interactions between the BH3-binding groove of cellular antiapoptotic BCL-2 relatives and a BH3-domain containing peptide or fragment [21,22,36]. For a detailed structural comparison, we used the crystal structure of BCL-X_L_ in complex with BAD that we have determined to 2.3 Å resolution (Table 1), in which 27 residues of BAD bound to BCL-X_L_ as an extended α-helix and all the rest of the residues were totally disordered. A sequence alignment based on the structural comparison showed that four out of five residues within proapoptotic BH3 domains that are critical for their interactions with the BH3-binding groove [21] are conserved as Leu110, Leu114, Asp119 and Phe121 in Beclin1 (Figure 2A and 2B). The remaining residue, which is isoleucine or methionine in the BH3 domains, is substituted as Thr117 in Beclin1. These five residues occupy spatially and chemically equivalent positions at the BH3-binding groove of M11 as the corresponding residues of BAD bound to BCL-X_L_ (Figure 2A). Additional structural comparison involving the BCL-X_L_–BAK, BCL-X_L_–BIM and MCL-1–BIM complexes led to the same conclusion, as the five residues are conserved in the BH3 domains of BAD, BAK and BIM (Figure 2B) and they occupy the equivalent positions at the BH3-binding groove of BCL-X_L_ or MCL-1 (Figure S2). The side chain hydroxyl group of Thr117 of Beclin1 is situated in a hydrophobic milieu, and therefore this residue appeared to make an insignificant or adverse contribution to the helix-groove interaction, in contrast with isoleucine or methionine in the canonical BH3 domains. Thr117 is conserved in the Beclin1 orthologues of vertebrates, but not in those of lower organisms (Figure 2C). Threonine for this position might have been chosen to tune the affinity of Beclin1 for cellular BCL-2 or BCL-X_L_ at a physiologically optimum level. Another noticeable difference from the canonical BH3 domains is that the Beclin1 α-helix has a hydrophobic patch composed of Val116, Leu120 and Ile123 that are not shielded by the BH3-binding groove (Figures 2D and S3), while those of other BH3 domains, including that of BAD (Figures 2D and S3), are distinctively amphipathic. The exposed hydrophobic residues of Beclin1 are identically or similarly conserved throughout species (Figure 2C), suggesting that they may play an as yet unknown important role. These structural and sequence comparisons indicate that Beclin1 has an atypical BH3 domain characterized by the threonine substitution and the exposed hydrophobic patch.

### M11 Interacts with Beclin1 Much More Tightly and Inhibits Autophagy More Potently than Cellular BCL-2

**Figure 3:**
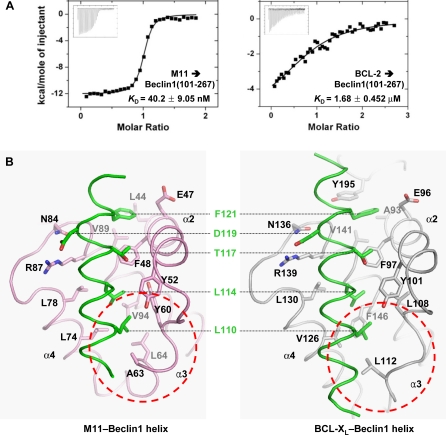
M11 Interacts Much More Tightly with Beclin1 than BCL-2 and BCL-X_L_ (A) ITC analysis. The measurement was carried out by titrating 0.1 mM of M11 or BCL-2 into 5 μM of the indicated Beclin1 fragment. The *K*_D_ values were deduced from curve fittings of the integrated heat per mol of added ligand. (B) M11–Beclin1 interface is tighter than that of BCL-X_L_–Beclin1. The structures of M11–Beclin1 fragment (left) and BCL-X_L_–Beclin1 peptide (right) are compared side by side. In both the structures, the five consensus BH3 residues of the bound α-helices and the side chains of M11 or BCL-X_L_ interacting with those residues are shown as sticks and labeled. Noted are the tighter interactions of the N-terminal region of Beclin1 with the α3 helix of M11 than the corresponding region in the BCL-X_L_–Beclin1 helix (indicated by dotted circles).

In contrast to the robust interaction between M11 and Beclin1(101–267), we found that BCL-2 interacts with Beclin1(101–267) weakly with a *K*_D_ of 1.7 μM (Figure 3A), which is similar to the *K*_D_ value (1.1 μM) for the interaction between BCL-X_L_ and a Beclin1 peptide [32]. In order to account for the huge difference in the binding affinity, we compared our structure with the BCL-X_L_–Beclin1 peptide structure [32]. Compared with 950 Å^2^ interface of BCL-X_L_ buried by 22 residues of Beclin1, the binding interface of M11 is smaller (860 Å^2^) and involves fewer Beclin1 residues (a total of 16 residues). However, the binding surface of M11 renders tighter hydrophobic interactions with Beclin1 compared with that of BCL-X_L_ (Figure 3B). For example, while Phe121 of Beclin1 interacts with Ala93 of BCL-X_L_, it interacts with the corresponding but bulkier residue Leu44 of M11 (Figure 3B). Another notable difference is that the bound Beclin1 helix interacts tightly with the α3 helix of M11, while it interacts poorly with the corresponding region in BCL-X_L_ (Figure 3B), which consistently exhibits poor electron density (Figure S4) and high temperature factors [32]. As a result of these and other differences in the binding interactions, the M11–Beclin1 helix makes 88 intermolecular carbon-carbon contacts (distance < 4.2 Å), while the BCL-X_L_–Beclin1 helix makes 76 such contacts, indicating that the marked difference in the binding affinity arises from the difference in the shape complementarity, and thus the quality, of the hydrophobic interactions.

**Figure 4:**
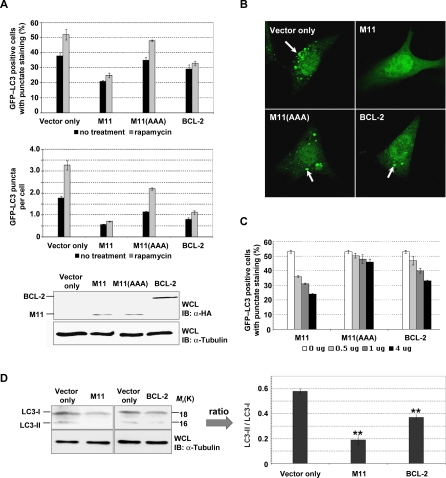
M11 Inhibits Autophagosome Formation in NIH3T3 Cells More Efficiently than BCL-2 (A) Light microscopic quantification of autophagy. After transfection with a GFP–LC3 expression plasmid together with the vector encoding the indicated protein, cells were maintained under normal conditions or treated with 2 μM rapamycin for 4 h. M11(AAA) is an M11 mutant containing three alanine substitutions at the BH3-binding groove. Autophagy was quantified as the percentage of GFP–LC3 positive cells (top) or as the number of autophagosomes (GFP–LC3 positive dots) per cell (bottom). The expression of M11 resulted in fewer GFP–LC3 positive cells or spots than the expression of BCL-2. Data represent mean ± s.d. of three experiments. Expression levels of M11, M11(AAA) and BCL-2 are shown below. (B) Confocal microscopic images of the rafamycin treated cells. GFP–LC3 was detected using an inverted fluorescence microscope. Arrows indicate autophagosomes labeled with GFP–LC3. (C) Dose response. NIH3T3 cells were transfected with GFP–LC3 expression plasmid together with increasing amount of plasmid encoding the indicated proteins. At 16–18 h posttransfection, cells were subjected to 2 μM rapamycin treatment for 4 h and autophagy level was quantified as described at (A). (D) LC3 mobility shift. The whole cells lysates of the rafamycin treated cells were subjected to immunoblotting with anti-LC3 and anti-tubulin antibodies (left). The ratio of quantified band intensities is also shown (right). The cleaved form (LC3-II) of the LC3 precursor (LC3-I) was undetectable and the level of LC3-II/LC3-I was far lower in the cells expressing M11 in contrast with the cells expressing BCL-2. Data represent mean ± s.d. of three experiments. **, *P* < 0.005 versus vector (Student *t* test).

To explore whether the marked difference in the binding affinity of M11 and BCL-2/ BCL-X_L_ for Beclin1(101–267) indeed correlates with their activity, we measured the autophagy-inhibiting capacity of M11 and cellular BCL-2. To quantify the level of autophagy, green fluorescent protein-tagged light chain 3 of microtubule-associated protein 1 (GFP–LC3) was used to indicate the formation of autophagosomes, which deliver cellular components to lysosomes for degradation and recycling during autophagy. GFP–LC3, a specific marker for autophagosome, moves from the perinuclear region into autophagosomal membranes under autophagy-promoting conditions such as starvation and rapamycin treatment [37,38]. In NIH3T3 mouse fibroblast cells, transiently expressed M11 inhibited autophagosome formation more efficiently than transiently expressed BCL-2, as evident from the rate of GFP–LC3 positive cells carrying autophagic vacuoles and the number of autophagosomes per cell, while the expression level of M11 was much less than that of BCL-2 (Figure 4A and 4B). The efficacy of M11 and BCL-2 was dose-dependent, as the ratio of autophagosome-carrying cells decreased with the increase of the amount of vectors used for transfection (Figure 4C). In these analyses, M11(AAA), the M11 mutant containing alanine substitutions of three conserved residues (S85A, G86A and R87A) within the BH3-binding groove [7] and barely able to bind Beclin1 [28], exhibited significantly reduced antiautophagic activity compared with the wild-type protein (Figure 4A, 4B, and 4C), suggesting that the Beclin1-binding capacity is essential for the antiautophagic activity of M11. To further compare their antiautophagic capacity, immunoblotting was also performed with an antibody against LC3. LC3-II, a cleavage product generated from the LC3 precursor (LC3-I), accumulates in the autophagosomal membrane during autophagy and therefore is widely used as a specific marker for autophagy processing [38,39]. In autophagy-inducing rapamycin-treated NIH3T3 cells, the overexpression of M11 suppressed the formation of LC3-II more efficiently than the overexpression of BCL-2 (Figure 4D). These data collectively demonstrate that M11 is a more potent autophagy inhibitor compared with cellular BCL-2, and that the potency directly correlates with their binding affinity for Beclin1.

### Interactions of M11 with BH3 Peptides of Proapoptotic BCL-2 Relatives

**Figure 5:**
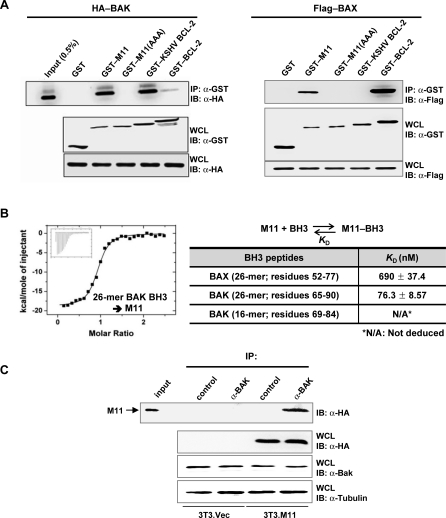
Analyses of the Interactions between M11 or BCL-2 Proteins and BAX/BAK (A) Cell-based binding assay. 293T cells were transfected with HA-tagged BAK or Flag-tagged BAX together with the indicated GST-tagged prosurvival BCL-2 proteins. Whole cell lysates were used for immunoprecipitation with anti-GST followed by immunoblotting with anti-HA or anti-Flag. While no band was detected for the interaction of GST–KSHV BCL-2 with Flag–BAX in this run, a faint band was detected in another run (Figure S5). (B) ITC analyses of the interactions of M11 with the BH3 peptides of BAX or BAK. The ITC analysis was carried out by titrating 0.1 mM of the indicated peptides into 5 μM of M11. The ITC run for the titration of the 26-mer BAK peptide is shown. The deduced *K*_D_ values are shown in the table. (C) M11 interacts with endogenous BAK. NIH3T3 cells were transfected with HA-tagged M11 and whole cell lysates were used for immunoprecipitation with control rabbit serum or anti-BAK followed by immunoblotting with anti-HA.

To gain insights into the antiapoptotic activity of M11, we analyzed the interaction between the apoptosis mediators BAX and BAK with M11. First, 293T cells were transfected with HA-tagged BAK or Flag-tagged BAX, together with each of four different GST-tagged prosurvival BCL-2 proteins including M11. These proteins, all in the full-length form, were transiently expressed. A following immunoprecipitation assay revealed that M11 exhibited a tight interaction with BAK (Figure 5A, left panel, lane 3) and a comparatively weak interaction with BAX (Figure 5A, right panel, lane 2). The M11 binding to BAX and BAK, as expected, depended on its intact BH3-binding groove, as triple mutations on the groove abrogated the binding interactions (Figure 5A). Definitely, the M11 binding to BAK was significantly tighter than the BCL-2 binding to BAK (Figure 5A, left panel, lane 6). However, the M11 binding to BAX appeared to be comparable at most or weaker compared with the BCL-2 binding to BAX (Figure 5A, right panel, lane 5). In this cell-based assay, KSHV BCL-2 also interacted strongly with BAK (Figure 5A, left panel, lane 5). However, its interaction with BAX was barely detected (Figure 5A, right panel, lane 4, and Figure S5), indicating that KSHV BCL-2 has much poorer affinity for BAX than M11. These results suggested that M11 could inhibit BAK strongly but BAX weakly and that the apoptosis inhibition by KSHV BCL-2 may not be through neutralizing BAX. Next, we quantified the interactions of M11 with 26-mer peptides containing the BH3 domain of BAX or BAK. In the analysis using ITC, M11 interacted with the BAX peptide weakly, exhibiting a *K*_D_ of 690 nM (Figure 5B). In contrast, M11 interacted much more tightly with the BAK peptide with a *K*_D_ of 76 nM (Figure 5B). These measured binding affinities explain and correlate with the cell-based binding assay using the full-length proteins of M11, BAX and BAK. We noted that 16-mer peptide (residues 69–84), shorter but spanning the BH3 domain of BAK, produced a flat titration curve and its binding affinity for M11 could not be deduced, and thus a longer BH3-containing sequence of BAK is required for tight binding to M11. In reflection of the binding assay, the interaction between M11 and endogenous BAK could be easily detected in NIH3T3 cells (Figure 5C).

**Figure 6:**
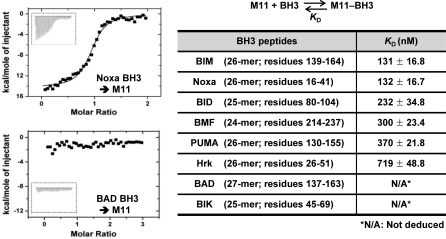
ITC Analyses of the Interactions of M11 with the BH3 Peptides of BH3-Only Proteins Representative ITC runs for the interaction of M11 with the Noxa and BAD peptides are shown, and the *K*_D_ values determined by this method are summarized in the table.

Also using ITC, we next analyzed the interactions between M11 and the BH3 domain-containing peptides of the eight well-studied BH3-only proteins BAD, BIK, BIM, BID, BMF, PUMA, Noxa and Hrk that act upstream of BAX/BAK. These BH3 peptides, containing 24 to 27 amino acids, are the same as or 1 to 2 residues longer than those used by Chen *et al.* for studying the interactions between the BH3-only proteins and a cohort of prosurvival BCL-2 proteins [40]. In their study, the long BH3 peptides did not appear to pose a problem of reduced helical propensities, because they bound to at least one of the BCL-2 proteins potently. Given this observation and the short BH3-binding groove of M11, which can be fully spanned by 19 residues of Beclin1 (Figure 2A), we conclude that the length of the BH3 peptides is likely to be optimal. As shown in Figure 6 and Table S2, M11 interacted with the BIM, Noxa, BID, BMF and PUMA peptides fairly tightly with the *K*_D_ values ranging from 131–370 nM, while it interacted with the Hrk peptide rather weakly (*K*_D_ of 719 nM). However, M11 did not interact or poorly interacted with the BAD and BIK peptides such that *K*_D_ values could not be deduced. Using an optical biosensor, Chen *et al.* previously quantified the interactions between the entire cohorts of the cellular antiapoptotic BCL-2 relatives with the BH3 domain peptides of the BH3-only proteins [40]. A comparison of these data with our results shows that M11 is dissimilar from any of the five cellular BCL-2 homologues in the selectivity and affinity for the BH3 domain peptides (Table S1). For example, while M11 has high affinity for the Noxa peptide but negligible affinity for the BAD peptide, BCL-2 exhibits the opposite binding affinity for the two peptides (Table S1). Importantly, M11 binds tightly the BH3 domain peptides of BIM and PUMA, which have potent cell-killing activity probably owing to their selectivity for all the five anti-death BCL-2 relatives [40]. Moreover, M11 exhibited extremely poor binding affinity for the BH3 domain peptides of BAD and BIK, which have limited selectivity for BCL-2/BCL-X_L_ and relatively poor apoptotic activity [40].

## Discussion

### Beclin1 Appears a Main Target of M11

A newly identified function of BCL-2 is the down regulation of autophagy through their inhibitory binding to Beclin1, which appears critical for cellular homeostasis [30]. As shown by others [32] and in this study, the BCL-2/BCL-X_L_ interaction with Beclin1 is quite weak compared with their interactions with the BH3-only proteins such as BAD and BIM [40]. The weak interaction explains the recent observation that endogenous BH3-only proteins induce autophagy by displacing Beclin1 from BCL-2/BCL-X_L_ [31]. Like the cellular kin, two viral BCL-2 proteins from γHV68 and KSHV are known to inhibit autophagy in addition to suppressing apoptotic death of cells [28,30]. In this study, we provided the structural basis for the inhibitory interaction of M11 with Beclin1, which is reminiscent of the canonical interaction between a BH3 peptide and a BH3-binding groove. Significantly, M11 bound to Beclin1(101–267) more tightly than BCL-2 did. Furthermore, the affinity of binding (*K*_D_ of 40 nM) between M11 and Beclin1(101–267) was higher than that between M11 and any of the ten different BH3 peptides used in this study. As a confirmatory experiment, we carried out a displacement test, where a complex between two proteins was challenged by another protein. Consistent with our affinity measurement, the M11–Beclin1(101–267) complex remained intact when it was incubated with the BIM, BID or Noxa peptide (Figure S6A). In contrast, the BCL-2–Beclin1(101–267) or BCL-X_L_–Beclin1(101–267) complex was easily disrupted by BAD or BIM peptide (Figure S6B). Conceivably, M11 could negate the proautophagic role of the BH3-only proteins under apoptosis-inducing conditions in contrast with BCL-2/BCL-X_L_. The observed robust interaction of M11 with the Beclin1 fragment, which correlates with its strong antiautophagic effect in NIH3T3 cells (Figure 4), suggests that Beclin1 may be a main target of M11 and that the inhibition of autophagy may contribute to the viral infection of cells.

Viral BCL-2 homologues, including M11, share limited sequence homology with the cellular kin [2]. Nonetheless, two available structures of KSHV BCL-2 and M11 have demonstrated that they are structurally homologous to the cellular kin and possess a prominent surface groove which binds the BH3 domain peptides from proapoptotic BCL-2 family members [7,41]. While the known BCL-2 homologues encoded by alpha and gamma herpesviruses exhibit only 20–30% overall sequence homology with each other [2], we noted that the residues of M11 significantly involved in the interactions with the Beclin1 fragment share 60–90% sequence similarity with the corresponding residues of the other herpesviral BCL-2 proteins (Figure S7). This observation raises a possibility that at least some alpha and gamma herpesviral BCL-2 homologues could interact with the BH3-like domain of Beclin1. In addition, some structural viral mimics of BCL-2, such as M11L of Myxoma virus [33] and N1 of Vaccinia virus [42], might also interact with Beclin1 through their BH3-binding groove.

### M11 Broadly Engages Proapoptotic BCL-2 Proteins

**Figure 7:**
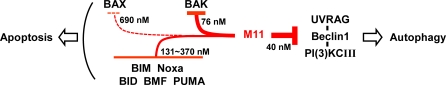
Model for M11 Action Mechanism The binding analyses presented in this study suggest that M11 antagonizes cell death by simultaneous inhibition of apoptosis and autophagy. The varied thickness of the arrows denoting the negative regulation by M11 indicates the inhibitory potency according to the *K*_D_ values determined in this study and shown next to the arrows. Dashed line is used to indicate that BAX may be inhibited by M11 but only weakly. Although not indicated in the figure, cellular BCL-2 proteins, protected by M11, may sequester and inhibit BAX (see text). PI(3)KCIII and UVRAG stand for class III phosphatidylinositol 3-kinase and UV irradiation resistance-associated gene, respectively.

The underlying mechanism of how viral BCL-2 homologues or mimics suppress apoptosis is not well understood. Perhaps M11L of Myxoma virus is best characterized in this regard. Through structural and biochemical analyses, M11L was shown to bind BAX, BAK and BIM proteins or peptides tightly but not the other proapoptotic BH3-only proteins [33]. Using a panel of M11L mutants containing an amino acid substitution at the BH3-binding groove, it was demonstrated that the prosurvival action of M11L largely depended on binding BAX and BAK [33]. The observation is consistent with a general expectation that viral BCL-2 would prefer to target BAX/BAK rather than the upstream BH3-only proteins [1]. In contrast with the binding selectivity of M11L, our quantitative binding analysis indicated that M11 primarily targets BAK, but not BAX, and broadly engages the BH3-only proteins except for BAD and BIK (Figure 7). How could M11, having the weak binding affinity for BAX, antagonize apoptosis of cells following the rise of the concentration of the activated BH3-only proteins under apoptosis-inducing conditions? We speculate that the neutralization of a subset of the BH3-only proteins (including BIM, BID, BMF, PUMA and Noxa) by M11 should prevent them from engaging their cellular prosurvival BCL-2 targets, and this protection would allow some fractions of the prosurvival proteins to keep suppressing the activation of BAX. This possibility is relevant to the suggestion that all the BCL-2 relatives keep BAX in check, whereas only BCL-X_L_ and MCL-1 inhibit BAK according to the so-called indirect activation model [18]. In this scenario, although M11 cannot neutralize BAD and BIK, MCL-1, having very low affinity for BAD and BIK [40], and other prosurvival protein molecules saved by M11 can inhibit BAX when M11 is expressed in the infected cell. An alternative possibility is that M11 inhibits the BAX activation by neutralizing BIM, BID, and PUMA, which are believed to directly activate BAX/BAK according to the hierarchical regulatory scheme [17]. Although further investigations may shed light on this important issue, the data presented here, including the weak interaction of KSHV BCL-2 with BAX (Figures 5A and S5), suggest that viral BCL-2 homologues may not necessarily target both BAX and BAK to suppress apoptosis.

### Concluding Remarks

We provided structural and biochemical bases for how M11 may subvert the antiviral host defense mechanisms, which is likely to involve both apoptosis and the Beclin1-dependent autophagy. Further studies are necessary to assess the importance of the Beclin1-dependent autophagy as an antiviral measure and to understand the consequences of the robust interaction of M11 with Beclin1 in the establishment and/or maintenance of the viral chronic life cycle. Our work provides a rational ground for future investigation to learn whether the inhibition of the Beclin1-dependent autophagy is the unique property of M11 and KSHV BCL-2 or is a general feature of other viral BCL-2 homologues or mimics.

## Materials and Methods

### Preparation, crystallization, and structure determination of the M11–Beclin1(101–150) complex.

The DNA fragments coding for M11 (residues 1–137) and mouse Beclin1 (residues 101–150) were cloned into pET30a (Novagen) and pPROEX HTa (Invitrogen), respectively. From these vectors, a two-promoter vector was constructed for coexpression of the two proteins. The protein complex was produced in E. coli BL21(DE3) strain (Novagen) at 21 °C overnight and purified using a Ni-NTA column (QIAGEN), a Hitrap Q anion exchange column (Amersham Pharmacia) and a Mono Q anion exchange column (Amersham Pharmacia), equilibrated with 20mM Tris-HCl (pH 8.0), 220mM NaCl and 1mM dithiothreitol. Crystals of the complex were obtained by the hanging-drop vapor diffusion method at 24 °C by mixing and equilibrating 1 μl of each of the protein solution (10 mg/ml) and a precipitant solution containing 25% (w/v) polyethylene glycol 3350, 0.2 M magnesium chloride, and 0.1 M imidazole (pH 7.0). Before data collection, the crystals were immersed briefly in a cryoprotectant solution, which was the reservoir solution plus 10% glycerol. A diffraction data set at 2.3 Å resolution was collected on the beamline 4A at the Pohang Accelerator Laboratory, Korea, and processed using the programs DENZO and SCALEPACK [43]. The structure was determined by the molecular replacement method with the *CCP4* version of MolRep [44] using the structure of M11 [7] as a search model. Subsequently, model building and refinement were carried out using the programs O [45] and CNS [46]. The final model does not include residues 1–4 and 136–137 of M11, and residues 101–105 and 125–150 of Beclin1, whose electron densities were not observed or were very weak.

### Preparation, crystallization, and structure determination of the BCL-X_L_–BAD complex.

The DNA fragment coding for mouse BCL-X_L_ (residues 1–196) was cloned into pPROEX HTa. This construct was used as a template for deletion mutagenesis to produce BCL-X_L_ lacking the internal long loop (residues 45–84) and the C-terminal tail region (residues 197–235). DNA fragment coding for mouse BAD (residues 43–204; corresponding to residues 1–168 of human BAD) was cloned into pET30a. A two-promoter vector was constructed from these two vectors. The protein complex was expressed in the E. coli BL21(DE3) RIG strain (Novagen) at 21 °C overnight and purified using a Ni-NTA column, a Hitrap Q anion exchange column and a HiLoad 26/60 Superdex 75 gel filtration column (Amersham Pharmacia), equilibrated with 20mM Tris-HCl (pH 8.0), 100mM NaCl, and 1mM dithiothreitol. Crystals of the complex were obtained by the hanging-drop vapor diffusion method at 4 °C by mixing and equilibrating 1 μl of each of the protein solution (5 mg/ml) and a precipitant solution containing 10% (w/v) polyethylene glycol 1000 and 10% (w/v) polyethylene glycol 8000. Before data collection, the crystals were immersed briefly in a cryoprotectant solution, which was the reservoir solution plus 16% glycerol. A diffraction data set at 2.3 Å was collected on the beamline 41XU at the Spring-8, Japan. The structure was determined by the molecular replacement using the structure of BCL-X_L_ [47] as a search model. The final model does not include residues 31–44 of BCL-X_L_, and residues 43–136 and 164–204 of BAD. Crystallographic data statistics are summarized in Table 1.

### Purification of BCL-2 family proteins and Beclin1 fragment.

Each of the DNA fragments coding for M11 (residues 1–137), mouse BCL-X_L_ (residues 1–44 and 85–196) or mouse Beclin1 (residues 101–267) was cloned into pPROEX HTa. A plasmid containing the DNA segment coding for human BCL-2 (residues 1–50 and 92–207) was also constructed. The resulting protein lacks the internal long loop (residues 51–91) and contains a replacement of residues 35–50 with residues 33–48 of BCL-X_L_, which was necessary for the solubility of the protein as reported earlier [48]. Each construct was introduced into the E. coli BL21(DE3) strain. The proteins were expressed at 21 °C overnight and purified using a Ni-NTA column and a Hitrap Q anion exchange column.

### Peptides.

Synthetic peptides of 25-mer (residues 101–125 of Beclin1), 20-mer (residues 106–125 of Beclin1), 16-mer (residues 69–84 of BAK), 26-mer (residues 65–90 of BAK), 26-mer (residues 52–77 of BAX), 27-mer (residues 137–163 of BAD), 25-mer (residues 45–69 of BIK), 26-mer (residues 139–164 of BIM), 25-mer (residues 80–104 of BID), 24-mer (residues 214–237 of BMF), 26-mer (residues 130–155 of PUMA), 26-mer (residues 16–41 of Noxa), and 26-mer (residues 26–51 of Hrk) were purchased from Peptron (Korea).

### Isothermal titration calorimetry.

All measurements were carried out at 25 °C on a MicroCalorimetry System (MicroCal). Protein samples were dialyzed against the solution containing 20 mM Tris-HCl (pH 7.4) and 100 mM NaCl. The samples were degassed for 20 min and centrifuged to remove any residuals prior to the measurements. Dilution enthalpies were measured in separate experiments (titrant into buffer) and subtracted from the enthalpies of the binding between the protein and the titrant. Data were analyzed using the Origin software (OriginLab Corp.).

### Autophagy analyses.

Autophagy was assessed by GFP–LC3 redistribution and LC3 mobility shift. For GFP–LC3 redistribution assay, NIH3T3 cells were transfected with a GFP–LC3 expression plasmid together with the vector encoding BCL-2, M11, or M11(AAA). At 16–18 h posttransfection, GFP–LC3 in the cells grown under normal and 2 μM rapamycin-treated medium containing 1% FBS for 4 h was detected using an inverted fluorescence microscope. The percentage of GFP–LC3-positive cells with punctuate staining was determined in three independent experiments. To quantify GFP–LC3-positive autophagosomes per transfected cell, six random fields representing 200 cells were counted. For the LC3 mobility shift assay, NIH3T3 cells transfected with the vector encoding BCL-2, M11 or M11(AAA) were treated for 30 min on ice, lysed with 1% Triton X-100 and then subjected to immunoblot analysis with an antibody against LC3 (Santa Cruz Biotech).

### Immunoprecipitation assay.

Each of fusion protein GST–BCL-2, GST–KSHV BCL-2, GST–M11 and GST–M11(AAA) was cloned into pcDNA5/FRT/TO (Invitrogen) and overexpressed in 293T cells together with HA-tagged BAK or Flag-tagged BAX. HA–M11, HA–M11(AAA) and HA–BCL-2 proteins were also cloned into pcDNA5/FRT/TO and overexpressed in NIH3T3 cells, respectively. Cells were harvested and lysed in NP40 buffer supplemented with a complete protease inhibitor cocktail (Roche). Immunodetection was achieved with anti-Flag (1:5000) (Sigma), anti-HA (1:5000), anti-GST (1:2000), anti-tubulin (1:1000), anti-BAK (1:100), or anti-Beclin1 (1:500) (Santa Cruz Biotech), which was incubated at 40 °C for 8–12 h. The proteins were visualized by a chemiluminescence reagent (Pierce) and detected by LAS 3000 (Fujifilm).

### Circular dichroism spectroscopy.

Data were collected on a JASCO model J-810 spectropolarimeter with a 0.2 cm cuvette. CD spectrum was recorded over the range of 200–250 nm in a nitrogen atmosphere with peptides dissolved in 40 mM sodium phosphate buffer (pH 7.0) containing 30% TFE at the concentration of 0.1 mg/mL. The spectrum was the accumulation of three scans corrected by subtracting signals from the buffer control. The law CD signal at 222 nm (in millidegrees) was converted to mean residue ellipticity ([*θ*]_obs_, in deg **^.^** cm^2^ **^.^** dmol^−1^) using the equation

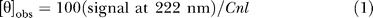
where *C* is the peptide concentration (in millimolarity), *n* is the number of residues in the peptide, and *l* is the pathlength (in cm). The contents of helix (*F*_helix_) was calculated using the equation

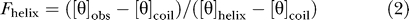
where [*θ*]_helix_ represents the mean residue ellipticity for a complete helix of infinite length at 0 °C (−42,500(1−3/*n*) deg **^.^** cm^2^ **^.^** dmol^−1^) and [*θ*]_coil_ is the ellipticity of a complete random coil at 0 °C (640 deg **^.^** cm^2 **.**^dmol^−1^) [49,50].

## Supporting Information

Figure S1 Helicity of Beclin1 Peptides The contents of α-helix of the indicated Beclin1 peptides in 30% TFE were calculated using the mean residue ellipticity at 222 nm acquired from the displayed circular dichroism spectra. The Beclin1(101–125) peptide has considerably higher helical contents than the Beclin1(106–125) peptide.
(257 KB TIF)

Figure S2 Interactions of BCL-X_L_/MCL-1 with BAK or BIM BH3 Domain BCL-X_L_ and MCL-1 are shown as surface models. The BAK and BIM residues shown in sticks are the five conserved residues in the BH3 domains. They occupy equivalent positions at the BH3-binding grooves as those in BAD (Figure 2A). The surface coloring scheme is the same as that of Figure 2A.
(4.4 MB TIF)

Figure S3 The Beclin1 α-Helix Bound to M11 Has a Hydrophobic Patch M11 and BCL-X_L_ are shown as surface models. The Beclin1 and BAD residues are shown as sticks and labeled. Three exposed hydrophobic residues of Beclin1 forming a hydrophobic patch on the opposite side of the BH3-binding groove are indicated by red ellipses and labels. In contrast, the bound BAD helix does not contain any exposed hydrophobic residue.
(3.6 MB TIF)

Figure S4 Interactions of the Beclin1 Helix (Shown in Green) with M11 (Left) or BCL-X_L_ (Right) The two views are shown in the similar orientation along with the final 2*F*_o_-*F*_c_ map (1 σ) at 2.3 Å and 2.5 Å, respectively. A portion of the Beclin1-binding interface involving the α3 helix (dotted circles) of BCL-X_L_ exhibits poor electron density in contrast with the corresponding region of M11.
(3.6 MB TIF)

Figure S5 KSHV BCL-2 Interacts with BAX Much Less Tightly than M11 293T cells were transfected with Flag-tagged BAX together with the GST–M11 or GST–KSHV BCL-2. Whole cell lysates were used for immunoprecipitation with anti-GST followed by immunoblotting with anti-Flag.
(215 KB TIF)

Figure S6 Displacement Test (A) Three BH3 peptides failed to displace Beclin1(101–267) bound to M11. After M11 was mixed with Beclin1(101–267) at 1:1 molar ratio and incubated for 1 h, the BIM, BID or Noxa BH3 peptide was added to the mixture and incubated for an additional 1 h. The M11–Beclin1(101–267) complex remained intact.
(B) BIM and BID peptides displaced Beclin1(101–267) bound to BCL-2 or BCL-X_L_. After BCL-2 and BCL-X_L_ were mixed with Beclin1(101–267) at 1:4 molar ratio and incubated for 1 h, the BAD or BIM BH3 peptide was added to the mixtures. The complexes between each of the BH3 peptides and BCL-2 or BCL-X_L_ appeared. The numbers above the gels indicate the final concentrations of the indicated proteins and peptides. The schematic drawings are shown to help to understand the experimental scheme and results.
(1.8 MB TIF)

Figure S7 Multiple Sequence Alignment The sequences of eight alpha and gamma herpesviral BCL-2 homologues are aligned (M11; KSHV BCL-2; RRV ORF16, rhesus rhadinovirus open reading frame 16; BHV4 BORFB2, bovine herpesvirus 4 BORFB2; HVS ORF16, saimiriine herpesvirus ORF16; MeHV BCL-2, meleagrid herpesvirus BCL-2; EBV BHRF1, Epstein-Barr virus BHRF1; EHV2 ORFE4, equid herpesvirus 2 ORF E4). The asterisk marks indicate the 11 residues of M11 that make hydrophobic contacts with the Beclin1 helix within 4.0 Å or hydrophilic contacts with Asp119 of Beclin1. The pink bars indicate the alpha helices in the M11 structure and the black bar indicates hydrophobic tails (HT). The residues exhibiting more than 70% of similarity are shaded.
(586 KB TIF)

Table S1 List of the Binding Affinities for the Interactions between the BH3 Peptides and M11 or Cellular BCL-2 Family Proteins The binding affinities for M11 are absolute values in *K*_D_ determined by ITC, while those for the other cellular BCL-2 proteins are relative values determined by competitive binding assays reported by Chen *et al.* [40]. Therefore, the binding profiles, not the binding affinities, have to be compared.
(125 KB DOC)

Table S2 List of Thermodynamics Values Determined by ITC for the Interactions between M11 or BCL-2 and the Indicated Fragment or Peptides (211 KB DOC)

### Accession Numbers

The coordinates of the M11–Beclin1 fragment structure and the BCL-X_L_–BAD structure have been deposited in the Protein Data Bank (http://www.rcsb.org/pdb/) with the accession codes 3BL2 and 2BZW, respectively. The accession numbers for the coordinates for the structures mentioned in this article are M11 (2ABO), BCL-X_L_ (1AF3), BCL-X_L_–Beclin1 (2P1L), BCL-X_L_–BAK (1BXL), BCL-X_L_–BIM (1PQ1), MCL-1–BIM (2NL9), and KSHV BCL-2 (1K3K). The National Center for Biotechnology Information (http://www.ncbi.nlm.nih.gov/) accession numbers for the protein sequences in the sequence databases are mouse Beclin1 (NP_062530), BCL-X_L_ (NP_033873), BAX (NP_031553), BAK (NP_031549), BAD (NP_031548), BIK (NP_031572), BIM (NP_997563), BID (NP_031570), BMF (NP_612186), PUMA (NP_573497), Noxa (NP_067426), Hrk (NP_031571), human Beclin1 (NP_003757), BCL-X_L_ (NP_612815), BCL-2 (NP_000624), MCL-1 (NP_068779), BAK (NP_001179), BIM (NP_619527), xlBeclin1 (AAH73292), trBeclin1 (NP_001032963), dmBeclin1 (NP_651209), scBeclin1 (BAA32104), M11(AAF19336), KSHV BCL-2 (NP_572068), RRV ORF16 (AAF59994), BHV4 BORFB2 (NP_076508), HVS ORF16 (CAA73630), MeHV BCL-2 (NP_073365), EBV BHRF1 (CAD53396), and EHV2 ORFE4 (NP_042601).

## Abbreviations

γHV68: murine γ-herpesvirus 68
BAD: BCL-2-associated death promoter
BAK: BCL-2-antagonist/killer 1
BAX: BCL-2-associated X protein
BCL-2: B-cell lymphoma-2
BH: BCL-2 homology
BID: BH3-interacting domain death agonist
BIK: BCL-2-interacting killer
BIM: BCL-2-interacting mediator of cell death
BMF: BCL-2-modifying factor
CCD: coiled-coil domain
CD: circular dichroism
ECD: evolutionarily conserved domain
GFP: green fluorescent protein
GST: glutathione-S-transferase
Hrk: harakiri
HT: hydrophobic tail
ITC: isothermal titration calorimetry
KSHV: Kaposi's sarcoma-associated herpesvirus
LC3: light chain 3 of microtubule-associated protein 1
MCL-1: myeloid cell leukemia sequence 1
PI(3)KCIII: class III phosphatidylinositol 3-kinase
PUMA: *p*53-upregulated mediator of apoptosis
TFE: trifluoroethanol
UVRAG: UV irradiation resistance-associated gene
