# Supplementary material for: Structural and Biochemical Bases for the Inhibition of Autophagy and Apoptosis by Viral BCL-2 of Murine γ-Herpesvirus 68
Source: PLoS Pathog. 2008 Feb 1;4(2):e25. doi: 10.1371/journal.ppat.0040025 (PMC2222952; doi:10.1371/journal.ppat.0040025)
Supplement: Supplementary file 1 [file ppat.0040025.st001.doc]

**Table S1. List of the binding affinities for the interactions between the BH3 peptides and M11 or cellular BCL-2 family proteins**

**Protein**

**Peptide**

**M11**

**BCL-2**

**∆C22**

**BCL-X_L_**

**∆C24**

**BCL-w**

**∆C29**

**MCL**

**∆N151**

**∆C23**

**A1**

**∆C20**

**BIM**

**131**

**2.6**

**4.6**

**4.3**

**2.4**

**4.3**

**Noxa**

**132**

**> 100,000**

**> 100,000**

**> 100,000**

**24**

**180**

**BID**

**232**

**6,800**

**82**

**40**

**2,100**

**9.3**

**BMF**

**300**

**3.0**

**9.7**

**9.8**

**1,100**

**1,800**

**PUMA**

**370**

**3.3**

**6.3**

**5.1**

**5.0**

**5.7**

**Hrk**

**719**

**320**

**3.7**

**49**

**370**

**46**

**BAD**

**N/A***

**16**

**5.3**

**30**

**> 100,000**

**15,000**

**BIK**

**N/A***

**850**

**43**

**12**

**1,700**

**58**

***N/A: Not deduced**

**Unit : nanomole**

The binding affinities for M11 are absolute values in *K*_D_ determined by ITC, while those for the other cellular BCL-2 proteins are relative values determined by competitive binding assays reported by Chen *et al* [40]. Therefore, the binding profiles, not the binding affinities, have to be compared.
