# Supplementary material for: Structural and Biochemical Bases for the Inhibition of Autophagy and Apoptosis by Viral BCL-2 of Murine γ-Herpesvirus 68
Source: PLoS Pathog. 2008 Feb 1;4(2):e25. doi: 10.1371/journal.ppat.0040025 (PMC2222952; doi:10.1371/journal.ppat.0040025)
Supplement: Supplementary file 2 [file ppat.0040025.st002.doc]

**Table S2. List of thermodynamics values determined by ITC for the interactions between M11 or BCL-2 and the indicated fragment or peptides**

**BH3 peptides**

***K*_D_ (nM)**

**∆H (kcal/mol)**

**-T∆S (kcal/mol)**

**∆G (kcal/mol)**

**BAX (26-mer; residues 52-77)**

**690 ± 37.4**

**-10.90 ± 0.14**

**2.49**

**-8.41**

**BAK (26-mer; residues 65-90)**

**76.3 ± 8.57**

**-15.10 ± 0.17**

**5.40**

**-9.70**

**BAK (16-mer; residues 69-84)**

**N/A***

**BH3 peptides**

***K*_D_ (nM)**

**∆H (kcal/mol)**

**-T∆S (kcal/mol)**

**∆G (kcal/mol)**

**BIM**

**(26-mer; residues 139-164)**

**131 ± 16.8**

**-20.18 ± 0.31**

**10.79**

**-9.39**

**Noxa**

**(26-mer; residues 16-41)**

**132 ± 16.7**

**-15.73 ± 0.25**

**6.35**

**-9.38**

**BID**

**(25-mer; residues 80-104)**

**232 ± 34.8**

**-12.26 ± 0.31**

**3.22**

**-9.04**

**BMF**

**(24-mer; residues 214-237)**

**300 ± 23.4**

**-6.34 ± 0.07**

**-2.56**

**-8.90**

**PUMA**

**(26-mer; residues 130-155)**

**370 ± 21.8**

**-11.97 ± 0.11**

**3.19**

**-8.78**

**Hrk**

**(26-mer; residues 26-51)**

**719 ± 48.8**

**-11.57 ± 0.11**

**3.19**

**-8.38**

**BAD**

**(27-mer; residues 137-163)**

**N/A***

**BIK**

**(25-mer; residues 45-69)**

**N/A***

**M11 + BH3	M11–BH3**

***K*_D_**

**M11 + BH3	M11–BH3**

***K*_D_**

***N/A: Not deduced**

***N/A: Not deduced**

**C**

**D**

**Beclin1 fragment / peptides**

***K*_D_ (nM)**

**∆H (kcal/mol)**

**-T∆S (kcal/mol)**

**∆G (kcal/mol)**

**Residues 101 - 267**

**40.2 ± 9.05**

**-14.33 ± 0.23**

**4.23**

**-10.10**

**Residues 101 - 125**

**99.0 ± 6.13**

**-14.57 ± 0.11**

**5.01**

**-9.56**

**Residues 106 - 125**

**1580 ± 98.3**

**-13.04 ± 0.27**

**5.13**

**-7.91**

**Beclin1 fragment**

***K*_D_ (nM)**

**∆H (kcal/mol)**

**-T∆S(kcal/mol)**

**∆G (kcal/mol)**

**Residues 101 - 267**

**1680 ± 452**

**-10.36 ± 2.08**

**2.48**

**-7.88**

**BCL-2 + Beclin1	BCL-2–Beclin1**

***K*_D_**

**M11 + Beclin1	M11–Beclin1**

***K*_D_**

**B**

**A**
